# Intrasexual Competition and Height in Adolescents and Adults

**DOI:** 10.1177/1474704917749172

**Published:** 2018-01-23

**Authors:** P. Polo, A. Fernandez, J. A. Muñoz-Reyes, M. Dufey, A. P. Buunk

**Affiliations:** 1 Laboratorio de Comportamiento Animal y Humano, Centro de Estudios Avanzados, Universidad de Playa Ancha, Valparaíso, Chile; 2 Grupo UCM de Estudio del Comportamiento Animal y Humano, Facultad de Psicología, Departamento de Psicobiología, Universidad Complutense de Madrid, Madrid, Spain; 3 Escuela de Psicología, Universidad de Santiago de Chile, Santiago de Chile, Chile; 4 Departamento de Psicología, Universidad de Chile, Santiago, Chile; 5 Department of Psychology, University of Groningen, Groningen, The Netherlands; 6 Faculty of Social and Behavioral Sciences, University of Curaçao, Willemstad, Curaçao

**Keywords:** intrasexual competitiveness, height, adolescence, adults

## Abstract

Intrasexual competition can be defined as the struggle between members of one sex to increase their access to members of the other sex as sexual partners. In our species, height is a sexually dimorphic trait probably involved in both intrasexual and intersexual selective processes. In the present research, we examined the relationship between height and individual differences in intrasexual competitiveness (i.e., the tendency to view same-sex interactions in general in competitive terms) in two populations of adolescents and adults of both sexes in Chile. According to our first prediction, among both adolescent and adult men, height was negatively associated with intrasexual competitiveness. In contrast, among women, height was not linearly nor quadratically related with intrasexual competitiveness as previously reported. Finally, adolescent men and women showed increased levels of intrasexual competitiveness compared to adult same-sex counterparts. Our results suggest that height is a relevant trait in mating competition among men. The lack of relationship between height and intrasexual competitiveness in women may suggest that the role of height in women mating competition may be more complex and mediated by other variables.

Sexual selection is the process proposed by Darwin to account for sex-differentiated traits that arise as a consequence of reproductive competition among members of one sex (Clutton-Brock, 2004). Same-sex individuals may compete to exclude rivals from mating leading to intrasexual selection of traits beneficial in contest competition (a mechanism commonly known as intrasexual competition), or they can compete to attract or charm individuals of the opposite sex leading to intersexual selection of traits that increase the likelihood of being chosen (a mechanism commonly known as mate choice; Darwin, 1871). Despite the usual terminology to refer to these two mechanisms of sexual selection, in the broad sense, intrasexual competition underlies both selective components of sexual selection (Andersson & Iwasa, 1996; Wiley & Poston, 1996). The relative success in this competition is an important component of fitness that may affect the perception of same-sex interactions as more or less competitive, resulting in individual differences in intrasexual competiveness, that is, the tendency to view same-sex others in competitive terms (Buunk & Fisher, 2009). Although height is a sexually dimorphic trait that has been related to both intrasexual and intersexual selection (reviewed in Stulp & Barret, 2016), its relationship with intrasexual competitiveness, measured with a psychometric test, has only been directly investigated in adult women (Buunk, Pollet, Klavina, Figueredo, & Dijkstra, 2009). In this study, we examined this relationship among men and women considering two age groups (i.e., adolescents and adults).

## Height and Intrasexual Selection

The role of height as a trait involved in intrasexual selection has been studied in terms of its association with physical strength, fighting ability, and aggressiveness, especially in men (Archer & Thanzami, 2007; Sell et al., 2009). Body size is an important feature that is associated with resource holding potential of individuals in many animals including humans (Parker, 1974; Sell et al., 2009). In accordance, there is evidence that taller men are physically stronger than smaller same-sex rivals and tend to have a better fighting ability (Archer & Thanzami, 2007; Puts, 2010; Sell et al., 2009; von Rueden, Gurven, & Kaplan, 2008), which may result in large repertoire of aggressive behaviors (Archer & Thanzami, 2007; Muñoz, Flores-Prado, Beltrami, Gil-Burmann, & Sánchez, 2009). In fact, since the first years of life, height or physical size is a variable that is relevant in dominance contests in both sexes (Pellegrini et al., 2007; Thomsen, Frankenhuis, Ingol-Smith, & Carey, 2011). Consequently, height may be considered as a signal of the competitive capacities of individuals involved in direct fights.

Women also compete to obtain and retain the best mates; however, they tend to avoid physical confrontations since physical damage could cause dramatic fitness costs (Campbell, 1999). Therefore, women tend to use more subtle mechanisms when competing with other women for a partner such as competitor derogation (Vaillancourt, 2013). In addition, the great differences in parental investment between sexes, with women investing more and having lower potential reproductive rates than men, lead to a higher incentive for men compared to women to compete at an intrasexual level (Clutton-Brock & Parker, 1992; Trivers, 1972). Sex differences in height and aggression, being women on average physically less aggressive and smaller than men, are thought to arise as a consequence of the mentioned difference in the intensity of intrasexual competition (Archer, 2009).

Among adults in Western societies, obtaining resources through physical aggression with same-sex rivals is not a commonly used strategy in social and professional interactions. However, it seems plausible that, at least in the mind of human males, the assessment of rival’s physical power, as a signal of their fighting ability and as an important factor in ancestral populations, affects their interactions and the degree of intrasexual competitiveness (Archer, 2009; Buunk, Pollet, Dijkstra, & Massar, 2011). In support of this argument, in naturalistic settings, there is consistent information that height affects social dominance in social interaction and respect from others (Stulp, Buunk, Verhulst, & Pollet, 2012, 2015).

## Height and Intersexual Selection

The role of height in *inter*sexual selection has also been explored in a large number of studies, for example, with respect to assortative mating on height (Stulp & Barret, 2016), the correlation between height and physical indices of attractiveness (like biceps circumference, see Archer & Thanzami, 2007), and sex differences in the preference for a taller or shorter mate (Courtiol, Raymond, Godelle, & Ferdy, 2010; Gillis & Avis, 1980; Pawlowski, 2003). Height of men is considered an attractive trait by women (Pierce, 1996). Although women’s preferences for men’s height are influenced by their own height, several studies have consistently found that women prefer men taller than themselves and with a height above men’s average in their population (Courtiol et al., 2010; Fink, Neave, Brewer, & Pawlowski, 2007; Stulp, Buunk, & Pollet, 2013). This is probably because height may signal both the capability of giving protection to the woman and the offspring and to extract limited resources from the social environment (Pierce, 1996). However, extremely tall men may be perceived as less attractive than tall men because their reduced reproductive success due to health problems (Nettle, 2002a). For women, there is contradictory evidence for the role of height in intersexual selection as some studies show that taller women are more likely to marry and to get higher educated husbands with better jobs (Smits & Monden, 2012), while other studies show that shorter females are preferred as mates (Shepperd & Strathman, 1989; Stulp, Buunk, Pollet, Nettle, & Verhulst, 2013). Furthermore, there is evidence that men tend to prefer women shorter than themselves but not too much shorter (e.g., Courtiol et al., 2010; Pawlowski, 2003; Stulp, Buunk, & Pollet, 2013). These results can be reconciled when we consider the evidence for a curvilinear relationship among women between height and various variables associated with mating such as reproductive success (Nettle, 2002b), jealousy (Buunk, Park, Zurriaga, Klavina, & Massar, 2008), and intrasexual competitiveness (Buunk et al., 2009). According to Buunk, Pollet, Klavina, Figueredo, and Dijkstra (2009), these curvilinear relationships with height are related to life history strategies, that is, women of intermediate stature are characterized by a slower life history strategy (with low levels of risk-taking, more foresight and planning, and persistence and self-directedness), whereas both taller and smaller women are characterized more by a fast life history strategy (accompanied by more jealousy toward rivals interfering in one’s relationship, an emphasis on mating effort, more risk-taking, etc.).

## Height and Reproductive Success

The evidence for the benefits associated with a larger but not extreme height in human societies, and especially among men, seems to put smaller men in a disadvantageous situation in intrasexual competition. Indeed, there is evidence supporting that jealousy, a proxy for both intrasexual competitiveness and mate guarding behavior, is more intensively expressed in smaller than in taller men (Brewer & Riley, 2009; Buunk et al., 2008). In addition, compared to smaller men, taller men are less sensitive to cues of dominance in other men (Watkins et al., 2010). In fact, smaller men may be in general accurate in their assessment, probably because they have to confront the potential devastating costs that an incorrect assessment of physical power of rival could have for them. In addition, in terms of mate choice, smaller men are in disadvantageous situation, as they are generally less attractive than taller men (Courtiol et al., 2010; Pierce 1996; Stulp & Barret, 2016).

On the other hand, for women, the relative benefits of height are less clear than for men. In contrast to men, women mainly compete within same-sex individuals in terms of attractiveness (Buss, 1989; Symons, 1995), instead of in terms of the capability to protect and obtain resources. Attractive women are more valued as allies by their same-sex peers and are more effective in derogating same-sex competitors (Buunk & Massar, 2014; Fisher & Cox, 2009), resulting in their superior position in terms of intrasexual competition. In addition, the expression of attractive traits in women is in part estrogen dependent and, as a consequence, it usually conveys information about health and fertility (Roney, 2009; Singh, 1993). Although some evidence suggests the opposite (e.g., Shepperd & Strathman, 1989; Smits & Monden, 2012), the available data suggest that females benefit from intermediate statures (Nettle, 2002b). Although the association is modest, taller and smaller females have less reproductive success due to health-related issues (Nettle, 2002b). This evidence suggests that men, who are mainly orientated toward cues of fertility (Buss, 1989), should show a preference for intermediate height in women.

## The Present Study

In the present research, we examined the relationship between height—assessed with an objective measure—and intrasexual competitiveness in two different groups, that is, adolescents and adults of both sexes in Chile. Although adolescents and young adults are close in terms of age, the social environments of both samples are different (high school vs. college), and more important, sexual relationships are not necessarily a common behavior in high school. Therefore, adolescents may face more competitiveness for the same sexually active peers (de Bruyn, Cillessen, & Weisfeld, 2012; Gallup, O’ Brien, & Wilson, 2011), despite that there is still much mating competition among 20- and 30-year-old (Wilson & Daly, 1985). We have measured intrasexual competitiveness through the Intrasexual Competition Scale (ICS; Buunk & Fisher, 2009). This is a reliable scale that was developed simultaneously in Canada and in the Netherlands to assess individual differences in intrasexual competitiveness. Specifically, this instrument measures the degree that each individual views the interactions with same-sex counterparts in competitive terms and implicates the desire to triumph over same-sex others, the desire to perceive oneself as better than same-sex others, envy and frustration when same-sex others are successful, and a feeling of malicious pleasure when the most successful lose confidence and hope. Although these are psychological attitudes reflecting general competitiveness, the instrument has been operationalized on dimensions relevant to mating or formulated in mating contexts (Buunk & Fisher, 2009).

In line with the foregoing evidence, we set out several sex-specific predictions. First, as shorter men are in a competitively disadvantageous position relative to taller men (Sell et al., 2009), we expect that intrasexual competitiveness will be negatively associated with height in both adolescent and adult men. However, since there is evidence that the benefits of being taller are reduced in extremely tall men (Nettle, 2002a), a quadratic relationship may also be expected. In addition, we expect increased levels of intrasexual competitiveness and a stronger negative association between intrasexual competitiveness and height among adolescent compared to adult men, as adolescence is a period in which competitiveness for mating opportunities through the use of aggression is prevalent due to the scarcity of sexually active peers (de Bruyn et al., 2012; Gallup et al., 2011).

Regarding women, since both shorter and taller women seem in a competitive disadvantageous position relative to women of medium height (Nettle, 2002b), we expect a quadratic relationship between intrasexual competitiveness and height in both adolescent and adult women. Following the same logic as for men, we expect increased levels of intrasexual competitiveness and a stronger negative association between intrasexual competitiveness and height among adolescent compared to adult women.

## Material and Method

### Participants

The adolescent sample was composed of 593 students from two high schools located in Santiago de Chile (303 women and 290 men) with ages between 16 and 18 years (*M* = 16.91, *SD* = 0.84 years). The adult sample included 246 adults (135 women and 111 men), with ages between 20 and 45 years (*M =* 27.80, *SD =* 6.06 years). They were volunteers for studies on psychology in one university in Chile. An ethical committee checked and approved the ethical compliance and consent forms used in the research. Both samples were recruited from public educational institutions and belonged to middle socioeconomic level.

### ICS

We applied a Chilean adaptation of the original ICS (Buunk & Fisher, 2009). This adaptation has been employed in previous studies (Fernandez, Muñoz-Reyes, & Dufey, 2014). The measure is composed of 12 items that assess the degree of competitiveness against same-sex peers (e.g., *I tend to look for negative characteristics in attractive women*). Answers are given on a 7-point Likert-type scale (1 = *not applicable at all* to 7 = *completely applicable*). The internal consistency obtained in the present study was similar to that observed in other studies (adolescents, α = .82, adults = α = .80, another Chilean sample from Fernandez et al., 2014, α = .85). The adolescent and adult samples differ in their variance in intrasexual competitiveness scores. That is, for both sexes, the *SD* in the adolescent sample is approximately twice as high as the *SD* reported in the adult sample. These differences are highly significant when we run Levene tests, men: *F*(1,393) = 53.36, *p* < .001; women: *F*(1,425) = 38.40, *p* < .001, which support splitting our sample in age groups.

### Height

This measure was obtained barefoot with a manual stadiometer in centimeters, although we express it further on meters.

### Statistical Analyses

To test for the predicted linear relationship between height and intrasexual competitiveness in men and for age-group differences in intrasexual competitiveness in men and women, we fitted two general linear models, one for men and one for women. We entered intrasexual competitiveness as the outcome variable, age and height as covariables, and age-group as a between-subjects factor with two levels (adolescents and adults). Then, we tested whether the inclusion of the interaction between age-group and height improved the initial models; thus, we assessed the predicted group differences in the relationship between height and intrasexual competitiveness. Finally, we entered into the initial models the quadratic terms of height to test for the quadratic relationship between height and intrasexual competitiveness.

Since polynomial equations can be highly affected by individual outliers (Cohen, Cohen, West, & Aiken, 2003), we employed the Tukey’s (1977) outlier labeling rule to identify potential problematic data. This procedure determines a lower and upper threshold according to the interquartile difference and a multiplier factor (*k*). Values below *Q*_1_ − *k*(*Q*_3_ − *Q*_1_) and above *Q*_3_ + *k*(*Q*_3_ − *Q*_1_) are considered outliers, being *Q*_1_ the first quartile and *Q*_3_ the third quartile. We employed the commonly value of *k* equal to 1.5. According to this procedure, we excluded two individuals from the adolescent men sample (*n* = 288), four from the adult men sample (*n* = 107), five from the adolescent women sample (*n* = 298), and six from the adult women sample (*n* = 129).

All the analyses were performed with IBM SPSS 21 statistical software and the overall level of significance was set up at *p* < .05.

## Results

**Figure 1.**
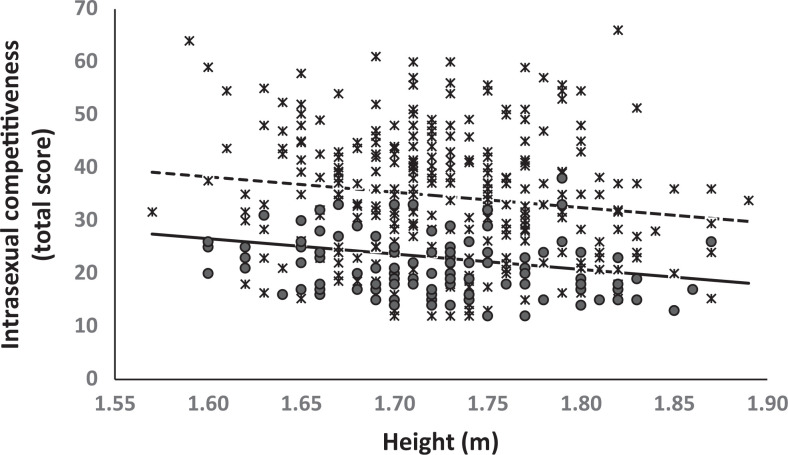
Relationship between height and intrasexual competitiveness in adolescent (dotted line and asterisks) and adult (solid line and circles) men. Data points (asterisks and circles) depict observed values and lines represent the expected values along the observe range of height according to a significant linear adjustment. The quadratic relationship was not significant and the expected values are not showed. Both adolescent and adult men showed a negative relationship between height and intrasexual competitiveness and adolescents showed increased intrasexual competitiveness compare to adults in any given height. When entered, the effect of the interaction between age group and height was not significant. See statistics in the text and in Table 2.

Descriptive statistics (means and standard deviations) for intrasexual competitiveness are presented in Table 1. The initial model for men (Table 2) shows that height was negatively related to intrasexual competition in both adolescents and adults, *B*_height_ = −28.90, *t*(391) = −3.30, *p* = .001, and adolescents showed higher levels of intrasexual competitiveness compared to adults, *B*_adolescents_ = 11.68, *t*(391) = 5.30, *p* < .001, Figure 1. However, the interaction between age-group and height was not significant when it was entered in the initial model, *B*_Adolescents × Height_ = −14.160, *t*(390) = −0.73, *p* = .468. This result indicates that the relationship between height and intrasexual competitiveness was similar in both age groups. Similarly, the inclusion of the quadratic term of height did not improve the initial model; both the linear and quadratic terms of height were not significant, *B*_linear_ = −88.22, *t*(390)= −0.23, *p* = .822; *B*_quadratic_ = 17.13, *t*(390)= 0.15, *p* = .880.

**Table 1.** Mean and Standard Deviation Split by Sex and Age Group of All Variables.

Variables	Men		Women	
	Adolescence (*n* = 288)	Adulthood (*n* = 107)	Adolescence (*n* = 298)	Adulthood (*n* = 129)
Intrasexual competitiveness	35.16 (11.78)	21.33 (5.62)	35.17 (12.87)	22.49 (7.43)
Height	1.73 (0.06)	1.72 (0.06)	1.59 (0.06)	1.60 (0.05)
Age	17.01 (0.89)	28.50 (6.00)	16.81 (0.77)	27.29 (6.09)

**Table 2.** General Linear Model Showing the Effect of Age-Group (Adolescents vs. Adults), Age, and Height on Intrasexual Competitiveness of Men.

Parameters	*B*	*t* Ratio	*p* Value	${\text{η}}_{\text{p}}^{\text{2}}$
Intercept	76.77	4.90	< .001***	.058
Age group = 1	11.68	5.30	< .001***	.067
Age	−0.20	−1.21	.228	.004
Height	−28.90	−3.30	.001***	.027

*Note.* Bold values indicate exact p-values. **p < .01. ***p < .001. Age group: 1 = adolescents, 0 = adults. Adults are the reference category. Outcome variable: intrasexual competitiveness.

**Figure 2.**
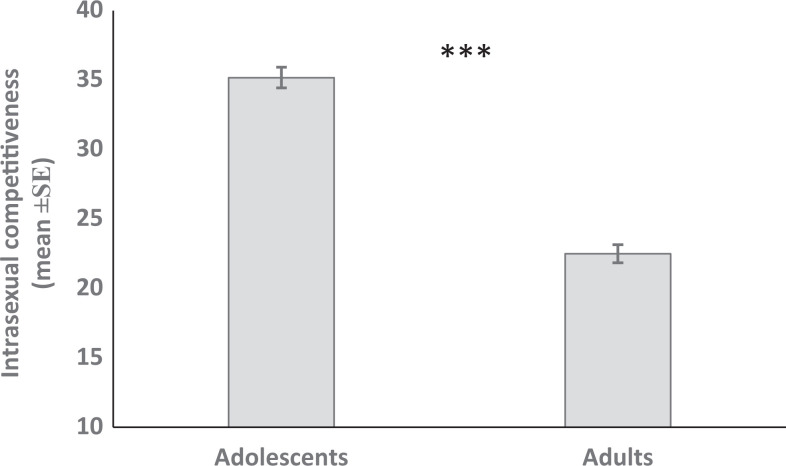
Bar plot of intrasexual competitiveness in women (mean ± standard error) according to age class. Adolescent women showed higher levels of intrasexual competitiveness than adult women. See statistics in the text and in Table 3. ***p < .001.

The initial model for women (Table 3) indicates that adolescents showed higher levels of intrasexual competitiveness compared to adults, *B*_adolescents_ = 10.64, *t*(423) = 5.05, *p* < .001, Figure 2, but no effect of height, *B*_height_ = 12.72, *t*(423) = 1.24, *p* = .214, nor age, *B*_height_ = −.20, *t*(423) = −1.20, *p* = .229, was found. In addition, the interaction between age-group and height was not significant when it was entered in the initial model, *B*_Adolescents × Height_ = −1.69, *t*(422) = −0.07, *p* = .945. Similarly, the inclusion of the quadratic term of height did not improve the initial model; both the linear and quadratic terms of height were not significant, *B*_linear_ = −653.20, *t*(422)= −1.39, *p* = .165; *B*_quadratic_ = 208.03, *t*(422) = 1.42, *p* = .157.

**Table 3.** General Linear Model Showing the Effect of Age-Group (Adolescents vs. Adults), Age, and Height on Intrasexual Competitiveness of Women.

Parameters	*B*	*t* Ratio	*p* Value	${\text{η}}_{\text{p}}^{\text{2}}$
Intercept	7.56	.44	.662	<.001
Age group = 1	10.64	5.05	<.001***	.057
Age	−0.20	−1.20	.229	.003
Height	12.72	1.24	.214	.027

*Note*: Bold values indicate exact p-values. ***p < .001. Age group: 1 = adolescents, 0 = adults. Adults are the reference category. Outcome variable: intrasexual competitiveness.

## Discussion

In the present study, we tested two sets of sex-specific predictions about the relationship between intrasexual competitiveness and height in two age groups: late adolescents and adults. Our results provide partial support for these predictions, showing the expected linear relationship among height and intrasexual competitiveness in men. However, among women, there were neither linear nor quadratic relationship between height and intrasexual competitiveness. In addition, we found support for the prediction that in both sexes, intrasexual competitiveness is higher among adolescents than among adults.

More specifically, we expected a negative relationship among height and intrasexual competitiveness in both adolescent and adult men. Our results clearly support these expectations as in both age groups we found that taller men were lower in intrasexual competitiveness. Taller men are generally physically stronger and better fighters than smaller same-sex rivals (Puts, 2010; Sell et al., 2009; von Rueden et al., 2008). Therefore, they will be more successfully than shorter men in contest competition over reproductive resources. In addition, taller men tend to be preferred by women (Courtiol et al., 2010; Pierce, 1996) and will outperform smaller men when competing in attracting mates. The association between height and success in intrasexual competition can explain our results, considering that the ICS can be regarded as a measure of the psychological predisposition to view interactions with same-sex individuals in a competitive fashion (Buunk & Fisher, 2009; Buunk, Fornari Bucksath, & Cordero, 2017). Thus, taller individuals may perceive the interaction with other men as less challenging because they are dominant and more attractive than smaller men. Conversely, smaller men will perceive the interaction with same-sex individuals as more threatening, especially in a mating context since they are in disadvantage in competitive terms. Our results seem to point toward an inverse relationship among the success in intrasexual competition and the attitude to view interactions with the same sex as competitive, but it remains to be tested whether this attitude is translated into higher frequency to exhibit intrasexual competitive behaviors. In other words, are smaller men behaving more intrasexual competitive to balance out their decreased success in this competition? This question requires future studies. Finally, no evidence of quadratic relationship among height and intrasexual competitiveness was found in this study. Benefits of being taller may be reduced on extremely tall men due to health issues (Shors, Solomon, McTiernan, & White, 2001) or decreased attractiveness (Hensley, 1994). Therefore, a quadratic effect of height on intrasexual competitiveness could be only present on those populations with higher average height in which the occurrence of extreme taller men is more frequent. Chile was placed in the 94-worldwide position according to men average height in a recent study (NCD Risk Factor Collaboration, 2016), accordingly, it is reasonable to think that the negative effect of extreme taller men is unusual in Chilean samples.

Our second prediction was partially supported as we found that adolescent men showed a higher level of intrasexual competitiveness than adults. Adolescence has been defined as both an age where intrasexual competition and reproductive activities are very salient (Gallup et al., 2011) and as an inflection point in the development of social status and reproductive trajectories (Ellis et al., 2012). Indeed, during adolescence, sexual activity emerges, and 31.9% of Chilean adolescents of 16 years old declare to be sexually active (INJUV, 2013). Therefore, extrapolating this national data to our sample, there may be more competition for sexually active peers (de Bruyn et al., 2012; Gallup et al., 2011). In contrast, among adults, the peak of sexual activity tends to occur among those between 20 and 30 years of age, with a considerable decline after the age of 30 (Dunson, Colombo, & Baird, 2002). In line with the foregoing, various studies have demonstrated a high intensity of physical intrasexual competition among adolescent men, as apparent in the display of risky behavior (e.g., Ellis et al., 2012) and in same-sex peer aggression (e.g., Gallup et al., 2011). Recently, Mailhos, Buunk, and del Arca (2017) found in adolescents a positive relationship between intrasexual competitiveness and overreporting one’s height. The authors noted that overestimation of one’s height could be interpreted as an indicator of status and physical dominance. Accordingly, our results indicate that adolescents perceive their same-sex social environment in more competitive terms than adults do, suggesting, in turn, that adolescents are prone to compete in a more intense manner over access to mates than adults. However, the relationships between height and intrasexual competitiveness present similar patterns in adolescents and adult men. That is, intrasexual competitiveness decreased to the same degree with increased height in both age groups, although adolescents showed higher levels of intrasexual competitiveness at a given height.

In contrast to our predictions, we did not find any effect of height on intrasexual competitiveness among women. That is, we were unable to replicate previous results showing a quadratic relationship among these two variables (Buunk et al., 2009). A possible explanation of our results can be found in the height distribution of both studies. In both our adolescent and our adult sample, women were relatively homogeneous in their height, compared to the sample reported in the Buunk et al. (2009) study (*SD* values: 5.46 in our sample vs. 6.27 cm). In addition, the quadratic relationship found among reproductive success and height is supposed to be caused by the poorer health of the tallest and smallest women (Nettle, 2002b). In short, apparently the quadratic relationship seems to appear only when the sample contains a wide range of heights. Future studies should test the relationship between height and intrasexual competitiveness in more diverse samples in terms of height. Nevertheless, there is evidence that taller women are less sensitive to cues of dominance in other females’ faces, and that women low in intrasexual competitiveness are less sensitive to cues of dominance (Watkins, Quist, Smith, DeBruine, & Jones, 2012). These results suggest that score on the ICS can be viewed as negatively related to dominance and height in both men and women, but in our study, this relationship was only found in men.

Finally, in women, we found a similar difference in the level of intrasexual competitiveness between adolescents and adults as in men. As adolescent women also compete with other women for mates, it is not surprising that intrasexual competitiveness is higher in adolescent than in adult women. This result supports a previous study performed in a Chilean sample that found a relationship between intrasexual competitiveness and age, with younger individuals showing higher intrasexual competitiveness (Fernandez et al., 2014).

Some relevant issues have to be considered as limitation in our study. In the first place, we have not measured relationship status in our sample. However, we did not consider this variable because previous studies found a negligible impact of relationship status on intrasexual competition mechanisms (see Fisher, Tran, & Voracek, 2008). However, when the differences were reported, these emerged for people who were married versus people who were either dating or single (Fisher, Cox, & Gordon, 2009). Because the nature of our sample, adolescent and college students, it seems reasonable to assume that most of them were single or dating and only a minor proportion could be married. Still, future research must consider this dimension, especially when this will be performed in adult population. On the second place, we have no information about the participants’ socioeconomic status, which can affect the intrasexual competition from the use of familial wealth to attract a mate. But, as we have stated before, all participants were recruited from public educational institutions belonging to middle socioeconomic level. Although the Chilean education system is highly segregated and homogeneous in socioeconomic context (Elacqua, 2012), some individual differences can be present not considered on the present research. Finally, future research will benefit from the application of a longitudinal design to the study of the effect of height on intrasexual competitiveness in adolescents. Performing these studies considering early adolescence and including other physical and psychological variables can be especially useful for understanding the development of both individual and sexual differences in intrasexual competitiveness attitudes.

In conclusion, the present research underlines that height is a relevant trait to explain intrasexual competitiveness in men both in adolescence and in young adulthood. Accordingly, we like to suggest that taller men exhibit less intrasexual competitiveness because they are perceived by their potential rivals as more dominant, stronger, and more likely to win a fight. However, in women, no association between height and intrasexual competitiveness was observed. As our sample of women were relative homogenous in their height, this may indicate that only very tall and very small women will be in an inferior position in intrasexual competition to attract mates, and then, that only in samples or populations that show such variation the quadratic relationship will be observed. Finally, we like to emphasize that future studies are necessary, in different populations around the world, and preferably, considering the measure of intrasexual competitiveness in natural settings.
